# Migraine Visual Aura and Cortical Spreading Depression—Linking Mathematical Models to Empirical Evidence

**DOI:** 10.3390/vision5020030

**Published:** 2021-06-10

**Authors:** Louise O’Hare, Jordi M. Asher, Paul B. Hibbard

**Affiliations:** 1 Division of Psychology, Nottingham Trent University, Nottingham NG1 4FQ, UK; 2 Department of Psychology, University of Essex, Colchester CO4 3SQ, UK; jashera@essex.ac.uk (J.M.A.); phibbard@essex.ac.uk (P.B.H.)

**Keywords:** CSD, non-linear dynamic model, EEG/MEG, fMRI, GABA

## Abstract

This review describes the subjective experience of visual aura in migraine, outlines theoretical models of this phenomenon, and explores how these may be linked to neurochemical, electrophysiological, and psychophysical differences in sensory processing that have been reported in migraine with aura. Reaction–diffusion models have been used to model the hallucinations thought to arise from cortical spreading depolarisation and depression in migraine aura. One aim of this review is to make the underlying principles of these models accessible to a general readership. Cortical spreading depolarisation and depression in these models depends on the balance of the diffusion rate between excitation and inhibition and the occurrence of a large spike in activity to initiate spontaneous pattern formation. We review experimental evidence, including recordings of brain activity made during the aura and attack phase, self-reported triggers of migraine, and psychophysical studies of visual processing in migraine with aura, and how these might relate to mechanisms of excitability that make some people susceptible to aura. Increased cortical excitability, increased neural noise, and fluctuations in oscillatory activity across the migraine cycle are all factors that are likely to contribute to the occurrence of migraine aura. There remain many outstanding questions relating to the current limitations of both models and experimental evidence. Nevertheless, reaction–diffusion models, by providing an integrative theoretical framework, support the generation of testable experimental hypotheses to guide future research.

## 1. Introduction

Migraine is a debilitating disorder, yet there is little cross-discipline consensus as to its cause. Migraine is heterogeneous, consisting of several subtypes, the most common of which is migraine without aura (MO). However, of interest in the current review are those reporting migraine with aura (MA). These individuals fulfil the International Headache Society diagnostic criteria for migraine, but additionally experience hallucinations around the time of the onset of the headache. The majority of those with MA do not experience the hallucinations on every attack—79% of those with MA also experience attacks without aura [1]. Migraine aura is thought to be linked to a spreading wave of hyper-excitation (spreading depolarisation) across the brain’s surface followed by a period of reduced blood-flow (hypoperfusion) and suppressed neural activity (spreading depression) [2]. It is important to note that the wave of increased activity corresponds to the spreading *depolarisation*, while the suppressed neural activity corresponds to the spreading *depression*. Interestingly, the phenomenology of cortical spreading depolarisation and cortical spreading depression provides an insight into the probable mechanisms underlying migraine aura.

This review sets out to match models of migraine aura and the empirical evidence. However, linking the theoretical models of cortical spreading depolarisation and subsequent depression to experimental evidence in humans is challenging. Firstly, it is difficult to obtain direct electrophysiological recordings in humans during an attack due to their fleeting, unpredictable nature. Secondly, in order to model activity directly in humans, extremely invasive procedures would be required in order to capture the activity at the level of cortical columns and ion channels required by the model parameters. It is, however, possible to extrapolate from animal models to humans, although there are some important limitations that need to be born in mind. For example, along with the differences between human and animal brains, electrophysiological recordings in humans are on a much less fine-scale than those obtained from animals. Furthermore, while fMRI techniques record BOLD response, which are indicative of, but not a direct measure of, electrical activity in the cortex, it may be possible to relate these to the scale of electrophysiological recordings in humans. Finally, the evidence and knowledge about migraine triggers and behavioural performance may provide some insight into the level of excitation and inhibition in the brain. Although this may be on a different level of abstraction to the level of neurotransmitters, it could still be related to the parameters of a model that seek to provide an understanding of migraine aura. While it is currently not possible to draw definitive conclusions, by combining the weight of evidence for what is known about migraine aura phenomenology, triggers, and electrophysiological evidence, in this review, we seek to draw links between theoretical models of migraine aura and the precise physiological mechanisms to which these models correspond. Effective theoretical models could be used to help combine diverse experimental findings from reported phenomenology, electrophysiology, and behavioural performance and thus better understand migraine aura. However, the multi-disciplinary nature of the study of migraine means that many models are not accessible to those working in particular domains. The advantage of using these particular models is the flexibility to include abstract elements that mirror the attributes of physical features. These layers of abstraction can, for example be on the level of neurotransmitters or on the level of oscillatory activity related to measured EEG activity. This means that the right level of abstraction can lead to testable hypotheses to better demonstrate the mechanisms in migraine aura.

The aims of this review are (i) to provide an accessible introduction to these models and demonstrate how they can test hypotheses related to migraine, (ii) to connect how the experimental evidence may support them in the case of migraine, and (iii) to highlight the benefits of using these flexible models to obtain insights into the mechanisms of migraine and migraine-aura.

### 1.1. Phenomenology of Hallucinations and Visual Aura

A hallucination is said to occur when an observer perceives a sensory event in the absence of an external stimulus [3]. Migraine aura is a hallucinatory experience that can occur in any modality. At least 57% of people with migraine aura are thought to experience this visually, [4], and this may be as much as 98% [5].

**Figure 1:**
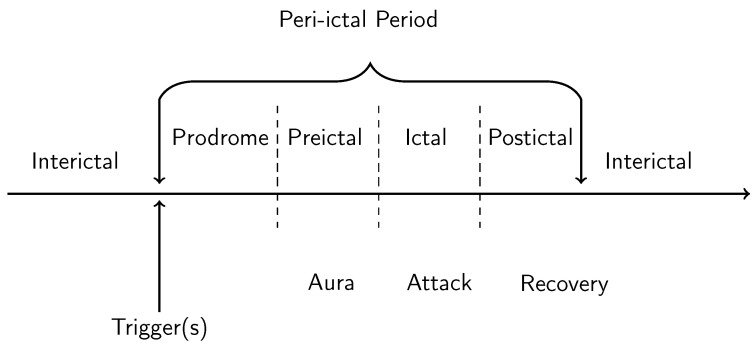
Time course of the stages of migraine.

Migraine aura consists of hallucinatory experiences that tend to appear shortly before the onset of the headache itself; the aura symptoms preceding headache onset on average by around 10 min [1] (see Figure 1). The duration of the migraine aura is variable, typically lasting between 5 min and 1 h [6], with the average duration being around half an hour [1]. However, there is also the phenomenon of prolonged migraine aura, which can last over one hour to several days [7]. Hemiplegic migraine aura, which involves weakness of the body, tends to last for longer than one hour [6]. There have been several studies documenting the quality of migraine aura hallucinations, although it must be noted that these vary considerably between individuals [8]. Aura hallucinations are sometimes unilateral, sometimes bilateral; sometimes on the same side as the headache, and sometimes not [8]. The most common symptoms of visual migraine aura are the positive symptoms of flashes of light, “foggy” vision, zig-zag lines, flickering lights, and the negative symptom of a scotoma, a temporary blindness in an area of the visual field) [9]. One of the most typical hallucinations is the “teichopsia”, or “fortification spectrum” which is best described as a “zig-zag” pattern [10]. Illustrations of fortification spectra have been documented by a range of authors, e.g., [11,12,13], and they are an important tool in discriminating migraine from other disorders. Finally, while there are also more complex hallucinations such as the perception of people and objects, these tend to be idiosyncratic and less common than zig-zags patterns, lines, flashing lights and scintillating scotoma [8,9] and are not considered in the current review.

The individual elements of hallucinations can help discriminate migraine aura from other disorders, such as the typically reported coloured discs in occipital epilepsy [14,15] or the shorter duration of hallucinations in visual epilepsy that occur without precipitating triggers [16]. Isolated attributes of the hallucination alone are not, however, a reliable means for an accurate differentiation between migraine and other disorders, which requires that the entire set of hallucinations and the duration of their effects are taken into account [17], alongside the other defining characteristics of migraine.

The phenomenological qualities of visual hallucinations have been used to generate theoretical models of the mechanisms of, for example, occipital epilepsy [14,15], Basilar Artery Migraine (BAM) aura [18], drug-induced hallucinations [19], and hypnagogic hallucinations [20,21]. Thus, understanding how the specific qualities of the migraine aura correlate with physiological measures in the brain may give insights into the reasons why these symptoms occur and thus provide insights into regarding the nature of migraine itself.

The aura can start in either central or peripheral vision [8,22]. One case study reported on an individual [23] who had recorded their migraine aura for many years. This revealed that their aura predominantly started in the central visual field but on occasion started in the periphery. Whilst the aura was on one side, it could start in either hemifield. Migraine aura is restricted to one or the other visual hemifield in many people, although it can also be bilateral [8]. fMRI evidence has shown that migraine aura stops at certain boundaries, corresponding to the sulci [24,25].

### 1.2. Cortical Spreading Depression as the Proposed Physiological Correlate of Aura

**Figure 2:**
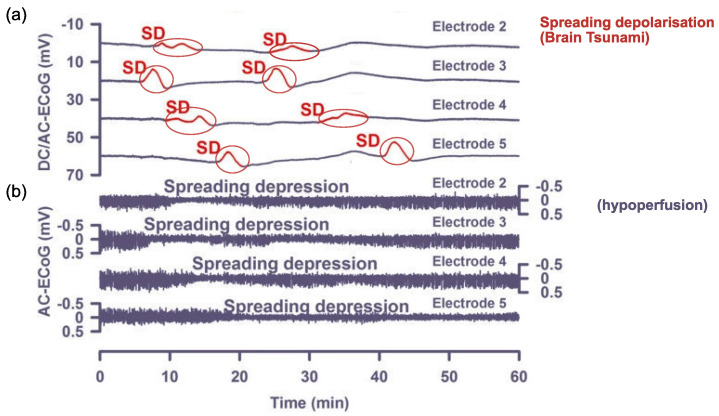
To visualise the electrical activity found in cortical spreading depression, we present an excerpt from Dreier et al. [27] (Figure 3: reproduced under the terms of a Creative Commons Attribution Non-Commercial License) of an intracranial recording of spreading depolarisation and spreading depression that was recorded in a terminal patient prior to a stroke. (**a**) DC/AC activity showing the characteristically large depolarisation spreading across a period of an hour; (**b**) AC activity illustrating the depression of spontaneous activity caused by the large depolarisation.

Cortical spreading depolarisation and subsequent depression is proposed to be the neurobiological mechanism responsible for migraine aura. Cortical spreading *depolarisation* is characterised by a massive self-propagating wave of neural activity, informally called a “brain tsunami” [26], which is followed by a period of suppression of both spontaneous and evoked activity (*depression*) that propagates over the cortex. The initial wave of depolarisation can be many times the amplitude of normal spontaneous activity (shown in Figure 2a) and travels slowly at a rate of 2–5 mm per minute for 15 s or longer [27]. After this initial wave, there is a sustained hyperpolarisation of neurons and a reversal of the membrane polarity [28], which inhibits the generation of synaptic potentials. During this period of silence (suppressed spontaneous activity and reduced excitability), the cortical spreading depression propagates with the depolarisation wave. Recovery can take 5–10 min for spontaneous activity to begin to reemerge [29], but up to an hour [22] to return to normal levels. In contrast, evoked activity (e.g., as a response from a stimulus) takes longer, around 15 to 30 min, to recover [30,31].

The spatial and temporal features of the migraine aura have been shown to closely resemble those of cortical spreading depression [13,29]. The positive and negative hallucinations experienced in migraine aura are thought to be the result of cortical spreading depolarisation and depression [32], respectively. Specifically, the zig-zag patterns are thought to be due to the travelling wavefront of excitatory depolarisation, while the scotoma are thought to be the result of the subsequent depression of activity [32,33].

One of the earliest discoveries of cortical spreading depression was by Leao [30], who identified a reduction in the spontaneous activity after applying electrical stimulation to the rabbit, pigeon, and cat cortices [30,34]. However, obtaining electrophysiological recordings for spreading depression in humans requires subdural electrodes, which are usually only used after a traumatic brain injury (e.g., [35]. Owing to the difficulties and expense in obtaining human data, direct evidence of spreading depression has not yet been demonstrated during a migraine aura [28,36]. To date, non-invasive scalp-based EEG recordings have not captured evidence of spreading depolarisation [34,37], as the spatial resolution of EEG is not sensitive enough to detect the phenomenon. Evidence of spreading depression from animal studies has been used to model migraine aura in humans, allowing for closer examination of the mechanisms of altered cortical and subcortical excitation and inhibition [38]. The most common elementary hallucinations of migraine aura have been a starting point for mathematical modelling.

## 2. Models of Cortical Spreading Depression

There have been several attempts to model cortical spreading depolarisation and the accompanying hallucinations in general terms (not necessarily specifically for migraine); for a review, see Billock and Tsou [39]. It is important to note that many authors use the term “spreading depression” and “spreading depolarisation” interchangeably (e.g., [40]), as one is considered to follow the other [41]. In animal models reporting recording of the spreading depression, the authors acknowledge that a silent period follows a wavefront of strong activity [42]. Some authors explicitly acknowledge that the zig-zag patterns and other hallucinations are likely to be the result of the depolarisation wave, whereas if models are aiming to show the spread of the scotoma, then it is likely to be depression that is of interest [33]. These dynamic models of neural networks are based on reaction–diffusion equations such as the Wilson-Conwan Equation [43,44]. These models capture many of the important properties of the migraine aura. Firstly, they model both the positive phenomena, such as they appearance of zig-zag patterns, and the negative phenomenon of the scotoma, as waves of excitation and suppression, respectively. Secondly, they model the propagation of these waves across the visual field. Finally, they are also able to account for the fine-scale spatial structure of the zig-zag patterns. In this section, we outline reaction–diffusion models in general terms and how these have been used as models of cortical dynamics of the visual brain. We then review their role in explaining the hallucinatory experience of migraine aura and the factors that make networks of neurons more susceptible to hallucination in migraine with aura.

### 2.1. Reaction–Diffusion Models

Introduced by Turing [45], reaction–diffusion models take a theoretical approach to explaining behaviour and pattern formation [46] in biological, geological, and ecological phenomena.

For example, one of the more straightforward models, the Gray–Scott Equation [47], has been used to model pattern formation. This example includes two antagonist reagents, A and B, in a predator–prey-like relationship, modelled by a pair of linked differential equations. Here, two units of B could, for example, convert one unit of A into another unit B (the “reaction”), to model B consuming A. When the levels of A drop, through consumption, then so will the levels of B.

**Figure 4:**
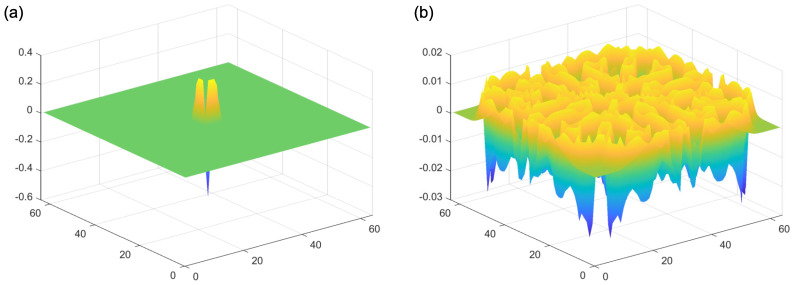
(**a**) Laplacian function for the initial condition of the activator, showing the direction and rate of the diffusion gradients. (**b**) Laplacian function for the activator at the end time point, showing the direction and rate of the diffusion gradients.

**Figure 5:**
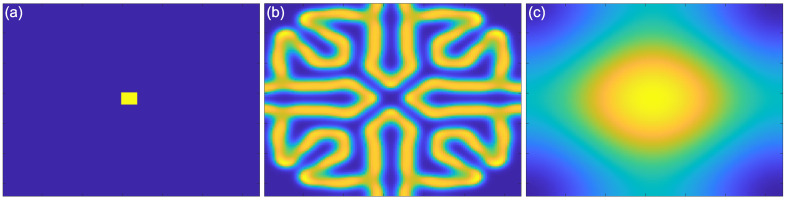
(**a**) The initial conditions of the activator component of the feed/kill rate model. Most of the area is zero, and there is a central peak seeded at 1, assumed to be due to random fluctuation. (**b**) The end point of the feed/kill rate model (5000 simulated seconds, with 4 iterations per second). The diffusion rate of the inhibitor (1) is double that of the activator (0.5). (**c**) The end point of the feed/kill rate model (5000 simulated seconds, with 4 iterations per second), when the diffusion rate of the inhibitor (1) is equal that of the activator (1).

In addition to this reaction component of the model, both reagents A and B spread out from areas of high to low concentration (“diffusion”). Importantly, these diffusion processes occur at their own independent diffusion gradients, determined by a two-dimensional Laplacian function. This function represents the spatial spread of the reagents A and B, from areas of high to low concentration. Examples of the Laplacian can be seen in Figure 4. An implementation of this kind of reaction–diffusion model can be found at: [48]. Critically, for the presence of self-emerging patterns, the diffusion rate of the inhibitor (A in this case) needs to be sufficiently large compared to the activator (B in this case). If this condition is met, patterns can be formed (Figure 5b). By contrast, the output of the model with equal diffusion rates of the activator and the inhibitor can be seen in Figure 5c. In this model, there is no periodic pattern formation. The initial conditions of the activator can be seen in Figure 5a.

**Figure 6:**
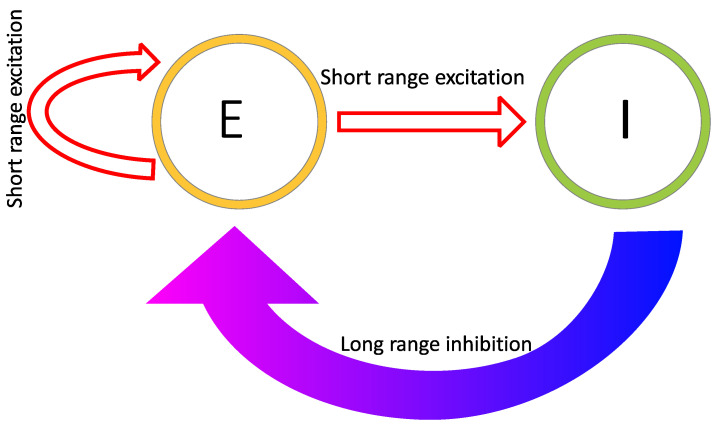
Figure on Activator (E) and Inhibitor (I) reagent levels. The activator E can increase levels of both itself and the inhibitor I, over the short range. The inhibitor reduces the levels of the activator over the long range.

**Figure 7:**
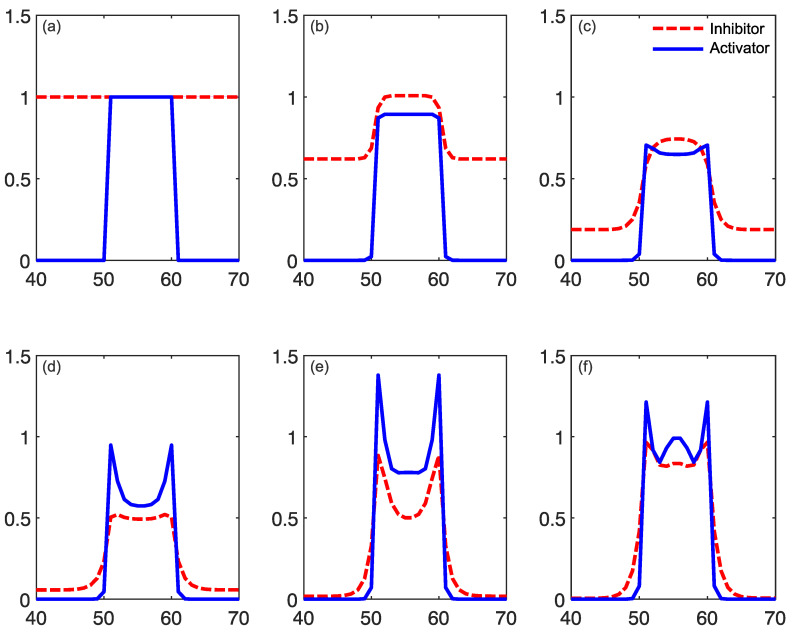
The reaction–diffusion model output for the initial conditions and the first few arbitrary simulated time stages. In this model, the diffusion rate of the inhibitor is twice that of the activator. (**a**) shows the initial conditions of the system, and the inhibitor is set to 1. The activator is initiated to zero, seeded with an area (“spike”) of activity due to random fluctuation. In this spike, the activator is set to 1. (**b**) The activator spike has enhanced the inhibitor in that area, so the inhibitor levels increase to greater than 1 (overshoot) in the region of the activator spike. The inhibitor in other areas is reduced, due to the decay rate. The inhibitor increase is slightly wider than the activator due to the different diffusion rates. (**c**) The effects of inhibition can be seen as the level of the activator reduces. (**d**) Inhibitor levels have fallen as there is less of the activator. The activator enhances both itself and the inhibitor, so at a certain point the levels of activator are low enough that the levels of inhibitor also drop. As the inhibitor has a faster diffusion rate than the activator, this means that the drop is faster for the inhibitor. As the inhibitor is reduced, the activator levels begin to rise again in response to the reduced inhibitor levels—note the edges of the peak, where the reduction in inhibitor is more pronounced and so the increase in activator is beginning to take shape. (**e**) The activator levels have risen in response to the reduced inhibitor levels, and this is more pronounced at the edges. Inhibitor levels start to rise again in response. (**f**) Levels of inhibitor rise again, completing the cycle, this time spatially extended from the original spike location. This continues to create a periodic pattern, under the right conditions.

Reaction–diffusion models include additional parameters, dependent on the specific model. In the Grey–Scott model, there are additional parameters for a “feed rate” for the introduction of the inhibitor A into the system, and a “kill rate” for the disappearance of the activator B out of the system. In the simpler versions of reaction–diffusion equations, these are constants. However, in more complex reaction–diffusion equations, these may be non-linear functions with multiple components. This tends to be the case in the reaction–diffusion systems representing brain activity, which have been used to model migraine aura. An excellent explanation is given in the work of Kondo and Miura [46], summarised here to illustrate how patterns can form and propagate through a network of this type. The activator increases the levels of *both* the activator and the inhibitor in the short-range, while the inhibitor reduces the level of the activator in the long-range (see Figure 6). Again it is important that the diffusion rate of the inhibitor B is sufficiently large in comparison to A in order for patterns to occur. An implementation of a model of this type, based on work by Gierer and Meinhardt [49], can be seen in Figure 7. In a particular area of the network, there is a random fluctuation resulting in slightly raised levels of the activator A (Figure 7a), which creates a feedback loop further increasing the levels of the activation in the area. The levels of the activator thus overshoot the original values. The levels of the inhibitor increase in response to this activation (Figure 7b) and reduce elsewhere through a process of diffusion and decay over time. The shape of the peak of inhibition is slightly lower at the edges, and the levels of the activator are reducde more in the centre compared to the edges of the area, resulting in a shape with a dip in the centre compared to the surroundings (Figure 7c). The activator enhances its own levels again, as these are higher in the edges, and this leads to a more pronounced dip in the centre (Figure 7d). The levels of the inhibitor are also enhanced in the edges compared to the centre. As the levels of the activator are higher in the centre compared to the surroundings, this shape is replicated by the inhibitor (Figure 7e), creating two peaks in both reagents with a dip in the centre. This relative release from inhibition in the centre compared to the surround allows the activator to increase again in the central region, and thus the cycle begins again and periodic patterns begin to form Figure 7f. The spatial constraints (Laplacian) and the diffusion rates are key to the pattern formation. If the diffusion gradients are equal, then the patterns will not occur. Additionally, the model depends on an initial random fluctuation to begin the self-emerging pattern formation.

### 2.2. Reaction–Diffusion Models and the Brain

In the case of the models relevant to the processes of the brain in migraine aura, the activator and the inhibitor represent the levels of excitation and inhibition at specific locations in a network of neurons, and the diffusion rates determine the overall speed of propagation of the travelling wave throughout this network. While these models can be defined on a relatively abstract level, their components have been linked directly to populations of neurons and their interactions. For example, Zhaoping and Li [50] has used a reaction–diffusion based model, called the V1 saliency hypothesis, to replicate human performance on visual search reaction times and performance on figure/ground segregation tasks. The V1 saliency hypothesis is based on a leaky integrate-and-fire model, a relatively common model of neuronal spiking. Zhaoping and Li [50] explicitly defines the units as coupled excitatory pyramidal cells and inhibitory interneurons and discusses the importance of ensuring that self-organising patterns of spontaneous activity, giving rise to hallucinatory perceptual experiences, do not occur.

One of the earliest models of the occurrence of hallucinations [21] used a pair of differential equations to represent excitation and inhibition, as a function of location in the cortex. Excitatory model units increase activity for both local excitatory and inhibitory units, and inhibitory units can reduce the activity of local excitatory and inhibitory units. The influence of each unit on the local activity is represented by a Gaussian weight to represent spatial interactions between cells [21]. The patterns emerge due to the organisation of the network of excitatory and inhibitory units. Tass [51] expanded the model to include a control parameter to allow for state switching, for example, to go from below hallucination threshold to hallucinating.

These models are based on the physiology of the cortex, and the rate of spread of the hallucinations [39], but they are a general model of hallucinations, not specific to the typical hallucinations of migraine. The earliest models of spreading depolarisation in migraine aura actually modelled the extracellular K+ levels (see [40] for a review), which might be considered to be related to cortical excitability. A later model by Reggia and Montgomery [52] included an inhibitor term, and this was able to capture the zig-zag pattern at the spreading depolarisation wavefront.

Later work by Dahlem et al., Dahlem and Chronicle [53,54] was aimed at modelling the hallucinations specific to migraine. This model was initially based on the assumption that the cortex is “weakly” excitable, meaning that the excitability levels must be within a certain range relative to threshold to trigger the attack, not much higher (otherwise there will be constant attacks) or much lower (in which case attacks will never trigger) [55]. This susceptibility term is represented in the non-linear function specifying the reaction rate of the inhibitor, defined in [41].

The models aim to replicate the mechanisms of the hallucinations, and they have captured some aspects that relate to the perceptual experience. The models incorporate threshold excitability levels to capture the switching of state of behaviour (bifurcation) from not having an attack to triggering the attack, as in the work introduced by [51]. General models of hallucinations have been able to recreate reported patterns, including spirals and concentric circles [21]. Tass [51] later developed a version able to capture dynamic hallucinations as well (e.g., “blinking rolls”) [51]. Terms are also included to represent the propagation boundary conditions in order to capture the behaviour of the travelling wave of excitation itself, namely whether it collapses in on itself or continues through the cortex. In the case of migraine, Dahlem and Hadjikhani [41] successfully replicated the scotoma, and the speed of its propagation over the cortex matched the hallucination expansion.

The activity modelled by reaction–diffusion models depends on the excitatory and inhibitory connections between nodes in the network, which depend on their spatial relationship. Since early visual areas in the cortex are retinotopically mapped, there is a strong connection between the relative spatial positions of neurons and the locations in the visual fields that they represent. There will thus be a similar correspondence between the spatial properties of the reaction–diffusion models, defined by the physical locations of units relative to one another, and spatial position in the image. It is this correspondence that allows the models to capture the spatio-temporal properties of the aura experience.

Excitatory and inhibitory connections between units in models of the visual cortex also depend on features beyond the simple location of receptive fields, however. For example, connections that depend on both the location and orientation of features are central to association field models of contour integration [56] and models of visual saliency [50]. This encoding of orientation as well as position is built into the fine-structure of the mapping of properties across the visual cortex, in which clusters of cells encoding similar orientations (orientation columns) form a characteristic “pinwheel” pattern of variation in preferred orientation [57]. This regular organisation of the primary visual cortex for both position and orientation has been used to provide an explanation of the zig-zag appearance of the aura created by the propagation of the wave of depolarisation Dahlem and Chronicle [54]. This model includes a parameter called the “pinwheel spacing term” to quantify the spacing between orientation columns. By including this fine-scale architecture of the primary visual cortex, and taking into account the orientation tuning of neurons, this model provides a more detailed account of the quality of aura. This is important as it provides a link between the migraine experience, the known dependence of excitatory and inhibitory connections on both location and orientation, and the biological implementation of these functional connections in their spatial mapping in the visual cortex.

This model was able to recreate the zig-zag patterns of the fortification spectra at the leading edge of the scotoma, which is a hallucination specific to migraine. They were also able to recreate the reports that migraine aura tends not to engulf the entire cortex but extends as far as the periphery and then disappears [41]. This is because of the folding of the gyri in the brain. When the brain is convex, the travelling wave will accelerate. When the structure is concave, the travelling wave will decelerate. By considering the folded structure of the human brain, rather than thinking of the space as homogenous or flat, this aspect can be captured effectively.

While it may seem unintuitive to link abstract levels and the units of excitatory and inhibitory connections, these models were designed to capture pattern formation in various natural forms. Their strength lies in their ability to abstract the essential components of the network dynamics without needing to know the exact underlying mechanisms [46].

### 2.3. What Do Models Account for?

Reaction–diffusion-based models that are used to model visual hallucinations are different from simple feedforward convolution-type models of visual processing (e.g., [58,59]), as they do not all rely on an explicit image-forming *stimulus* input in the same way. Rather, they self-generate patterns of activity that correspond to visual hallucinations. The patterns emerge from the initial conditions and the properties of the network parameters, importantly, the ratio of diffusion rates between the activator and the inhibitor. This makes them useful in understanding the hallucinations of migraine aura, as in many cases there is no apparent reliable external trigger, but the aura is elicited when certain internal thresholds are reached.

The spatial mapping of the models is able to create a travelling wavefront that matches the speed of the progression of hallucinations across the visual field [53]. More recent models [41] have included complex susceptibility terms to recreate both the trigger threshold for the spreading depolarisation and subsequent depression, but also to understand the reason for the hallucination stopping in the periphery. Some models explicitly define lateral interactions (coupling) between units [60], whereas others use Laplacian functions to model these spatial aspects [61]. The Laplacian has been suggested to relate to the connectivity of the brain, which has been able to predict the spatial patterns and the natural frequencies of the oscillatory behaviour [61]. This is a relatively computationally efficient way of being able to model the connectivity for large areas of the brain. Connectivity is something that can be estimated from electrophysiological recordings, which will be discussed later in the review in Section 3.

Reaction–diffusion models have been applied to migraine aura and visual processing more generally in various ways. Some models *do* include a specific input stimulus, which can recreate the oscillatory response and resulting pattern formation from repetitive inputs [60], which may be helpful in relating to neural oscillations measured at the scalp. This will be discussed in more detail in Section 5. It is also possible to relate the behaviour of certain reaction–diffusion models to human performance on a visual task [62]. Orientation discrimination performance has been modelled using integrate-and-fire models [63]. Integrate-and-fire models can be considered a simplification of the Hodgkin–Huxley model, which is a model based on a set of reaction–diffusion equations. Seriès et al. [63] explicitly defined coupling between units and defined the spatial distribution using a wavelet function. Orientation tuning sharpening was effectively recreated by this model. The relationship of models to behavioural performance will be discussed in more detail Section 6.

There are multiple different models of migraine aura, as well as reaction–diffusion models for modelling visual task performance. The difference between those modelling aura and those modelling performance tends to be the input. The former aim to recreate the self-emergent patterns arising from the network properties themselves, while the latter focus on accounting for the response of a complex system to a particular stimulus. In order to initiate the self-emerging properties, stochastic (noisy) processes are needed to “seed” the initial reaction. This need not be an external signal; there is the potential for this to be internal to the system. With so many models and variants, it is important to focus on the most relevant ones. Dahlem and Hadjikhani’s [33] work on recreating the fortification patterns specific to migraine is an important step here. This group has successfully developed models recreating many of the aspects of migraine aura by including appropriate parameters. The next issue is what the parameters represent in terms of biological systems in the brain itself. Several suggestions have been made, and this will be covered in the next section.

## 3. Linking Models to Physiological Differences in Migraine

The models use terms defining excitation and inhibition, but in order to transition from the abstract model, these terms must have some physiological basis. Mechanical and electrical stimulation of the cortex can induce spreading depression in animal models, as can chemical methods such as introducing KCl and GABA [2], suggesting that the answer may be a complex interplay of several factors.

### 3.1. Ion Transfer

One suggestion is that these terms relate to the transfer of ions across the cell membrane. For example, the models of Dahlem et al. [53,54] suggest that their excitation term could represent the level of extracellular potassium ions (K+). Changes in the levels of extracellular K+ (both increase and decrease) have been shown to increase the chances of spreading depression in the chicken retina [64]. Sleep deprivation, a commonly reported migraine trigger (e.g., [65]), increases the extracellular K+ levels in animal models and also lowers the spreading depression threshold [66]. Additionally, changing from stationary to locomotion increases K+ levels [67], and physical activity can aggravate migraine [68].

Increased spike activity increases extracellular K+ [69]. Smith et al. [2] suggested the rapid increase in K+ following intense neural firing results in the propagation of spreading depression and that this might be controlled by NDMA [2]. Astrocytes seem to be key in protecting against the onset of the travelling wave [2,31], and these cells have an important role in K+ homeostasis [70]. Astrocyte density is lower in the visual areas of the brain, which might explain why spreading depression is relatively easy to trigger here [31]. However, K+ is not released before the onset of depolarisation of the cells, and glutamate release follows the K+ release, suggesting that these changes may be secondary to the onset of the attack [71].

K+ increase is only one aspect of the complex ion changes in spreading depolarisation and depression. As well as the increase in K+, the levels of Cl-, Ca2+, and Na+ all decrease [72]. However, K+ is of particular interest as it seems to be the key ion involved in CSD development—when the K+ threshold is reached, this initiates the CSD. Additionally, this can be prevented if the glial cells (which control K+ levels) are able to act [72].

A study involving introducing a pathological mutation (knock-in) into mice resulted in increased susceptibility to CSD, and this mutation specifically affected the Ca2+ channels [73]. As the Ca2+ channels have a role in controlling neurotransmitter release, particularly glutamate [74], this may be the mechanism of CSD. Additionally, these channels also have an indirect role in the release of the neurotransmitter GABA [75].

Estimating the involvement of specific ions is difficult as the change in concentration in other ions cannot be easily controlled in investigations of the spreading depression. For example, animal models show changes in Cl- concentration (an increase after an initial decrease) after repetitive stimulation in cat sensorimotor cortex [76]. It has been suggested that the ion transfer of the different ions are dependent on each other; for example, as K+ leaves the cells, Na+ ions exchange places, although the exchange with Na+ is not 1:1 [77]. Additionally, there is the issue of electrodiffusion [40]. As the ions are electrically charged, this will counteract any diffusion gradient unless the other (negatively) charged ions also move. Taken together, this makes it very difficult to isolate the ion of interest experimentally, and so although the most likely candidate seems to be K+, this is far from conclusive.

### 3.2. GABA

Neurotransmitters such as gamma-aminobutyric acid (GABA) could also be involved in the level of inhibition. Dahlem and Chronicle [54] suggest that GABA might be deficient in the cortex of those with migraine, and that extracellular K+ is the triggering factor for the attack itself. Increasing the level of GABA results in increased extracellular K+ [78]. However, a review of epilepsy suggests that GABA seems to affect Cl−, rather than extracellular K+ [79].

Using the GABA antagonist Metrazol, which disturbs the action of GABA, to activate the cortex in conjunction with 8–10 Hz photic stimulation resulted in spreading depression [80]. Those with MA taking a GABA agonist (sodium valporate) showed normalised performance on a psychophysical task (metacontrast masking) compared to those not taking this medication [81]. Sodium valporate also normalises transcranial magnetic stimulation (TMS) phosphene excitability [82]. Importantly, there are links between behavioural performance and GABA levels—in healthy participants, increased GABA levels relate to increased orientation discrimination specificity [83]. A more detailed discussion of behavioural tasks thought to rely on GABA concentration in those with migraine specifically will be discussed in Section 6 below. Spreading depolarisation can be aborted in the human cortex with the introduction of GABA [84].

In humans, some authors report that GABA concentration does not differ between migraine and control groups or between those with MA and MO [85,86]. However, others have shown lower levels of GABA, specifically in those with MA compared to controls [87]. It must be noted that these studies tend to have a relatively limited sample size due to the expensive methodology. Additionally, other authors reported that increased GABA was related to increased pain and severity [88], which does not support the idea that a lack of GABA is the problem in those with migraine. GABA agonists have shown some benefit in migraine, e.g., [89]; for a review, see [90]. However, according to a Cochrane Review, gabapentin, which increases GABA levels in the brain [91], does not help much for migraine prophylaxis [92].

### 3.3. Glutamate

Glutamate is another neurotransmitter related to migraine pathophysiology. Interictal glutamate levels in the occipital cortex have been shown to be higher in a mixed group of migraine with and without aura compared to controls [93]. Many studies estimate levels of combined glutamate and glutamine (Glx), and this combined measure is also higher in migraine compared to controls [94]. Interical MA showed higher levels of glutamate compared to controls, but glutamate levels did not correlate with VEP responses [95]. Findings on this are mixed, as other authors have shown no overall differences in glutamate levels, but did show a relationship between BOLD activity in response to chequerboard stimulation in those with MA [87]. It could be the case that specifically in MA, and specifically in the occipital areas, there are differences. Studies of this kind tend to have relatively small sample sizes due to their expensive nature, which decreases the reliability of statistical findings. A recent study showed that glutamate and GABA levels can correspond to occipital activation, but this depends on the current state—GABA correlated with brain activity under dark conditions, whereas glutamate levels correlated with the brain’s responses to chequerboard stimulation [83]. This study could help understand the mixed findings in previous research into glutamate and GABA in migraine—findings are likely to depend on the state of the visual brain at the time, whether processing information or resting. Many studies into the role of glutamate actually estimate the Glx combined measure. Glx contains both glutamate and glutamine. Glutamine is the precursor to both glutamate and GABA [96], so results must be interpreted with caution, as it is possible that there is a simultaneous increase in GABA levels due to the glutamine.

### 3.4. Electrical Stimulation

Direct electrical stimulation of the cortex has been shown to elicit spreading depression in animal models [30]. Intercranial strip recordings of migraine aura over the frontal areas have shown evidence of spreading depression, including a sharp increase in activity, followed by a “DC shift”. The DC shift indicates a reduction in activity compared to spontaneous levels of activity before the aura event [71]. A less invasive method of stimulating the cortex in human observers is to use transcranial direct current stimulation (tDCS), transcranial alternating current stimulation (tACS), or transcranial magnetic stimulation (TMS). Electrical stimulation (tACS) of the cortex can elicit phosphenes [97], but these do not tend to resemble the fortification spectra commonly reported in migraine [39].

### 3.5. Links between Models and Biology

**Figure 8:**
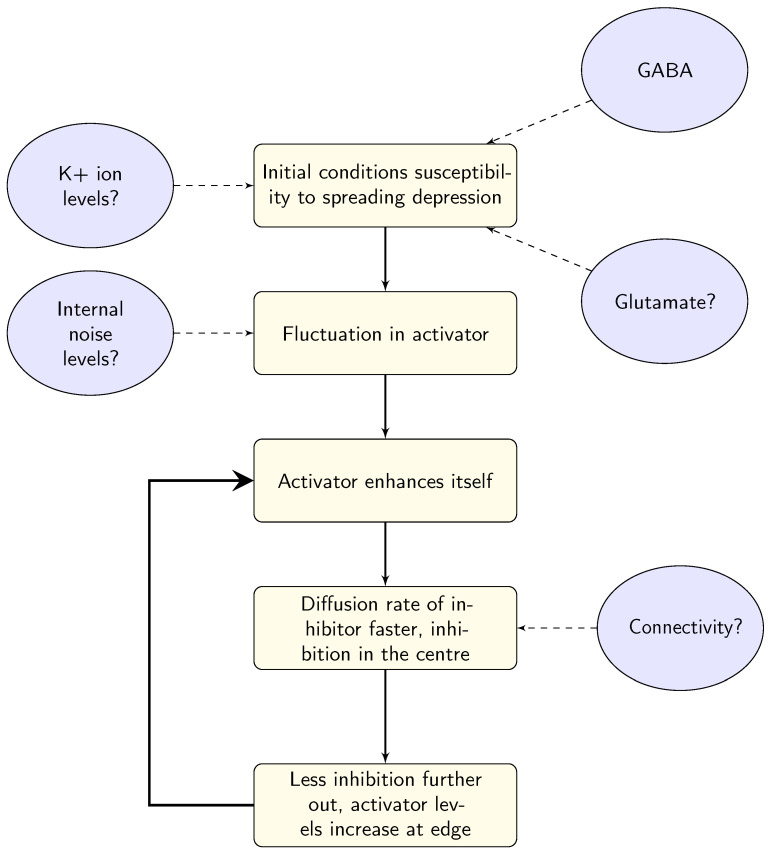
A schematic diagram of the main model stages and the possible parameters that have been associated with spreading depression. The initial conditions (prior to the initiating event) represent the susceptibility of the cortex to spreading depolarisation and subsequent depression. This could be due to neurotransmitters such as GABA, glutamate, and K+ ion levels. The next stage is the precipitating event, represented in the model by random fluctuation in activity. It is possible that random fluctuation is more likely in those with migraine due to increased internal noise in the brain. The next stage of the model is the self-enhancement of the activator. One of the key points for pattern formation is the ratio of diffusion rates for the activator and the inhibitor, which feasibly relate to the coupling strength or connectivity within the brain.

Linking the model parameters to biological systems in the brain is not a straightforward process. There are several suggestions for what the parameters in the models might represent, outlined schematically in Figure 8. At this stage, it seems that there is no agreed-upon answer, due to the complexity of both the models and the neural system, the expense of experimentally estimating levels of neurotransmitters, and most importantly the substantial variation between individuals with migraine. In order to gain some insights, large-scale longitudinal studies may be needed. Whilst these may pose logistical and expense issues for methods such as spectroscopy, there may be behavioural possibilities to investigate these differences. For example, Smith et al. [98] showed (indirectly) that it may be possible to increase levels of GABA through diet, demonstrating an effect on EEG responses over several sessions and an appropriate washout period. Using spectroscopy might be the gold standard for measuring neurotransmitter levels, but the cost of longitudinal studies of larger groups (to allow for individual variation) may make this prohibitively expensive. However, it may be possible to supplement these studies using behavioural techniques such as Smith et al. [98]. Additionally, the triggers of migraine could also give some insights into the system before the onset of the migraine attack.

## 4. Linking Models to Known Migraine Triggers

The factors that can precipitate a migraine attack may be particularly useful for understanding the onset of the attack and the migraine aura. A survey of 181 individuals with migraine reported that common triggers are stress, light, emotions, sleep disturbances, or alcohol useage [65]. A systematic review of migraine triggers, based on self-report in most studies, found the most commonly reported triggers to be stress (58% migraineurs), auditory stimuli (56%), fatigue (43%), fasting (44%), hormonal factors (females only, 44%), sleep disturbances (43%), changes in weather (39%), visual (38%) and olfactory (38%) factors, and alcohol (27%) [99]. Stress was also the most commonly reported trigger in several other studies [100,101,102,103].

### 4.1. Food-Based Triggers

Food is also commonly reported as a trigger for migraine; for example, red wine has been listed as a migraine trigger [103]. Red wine has anti-seizure effects by inhibiting firing rate through closing Na+ channels and opening Ca2+ channels [104], which would be thought to protect against spreading depolarisation and depression. However, given the complex interplay between ions and neurotransmitters, this may be an oversimplification of a complex system. Chocolate has also been reported as a trigger [103] by around 19% of participants [105]; however, studies trying to elicit migraine attacks using chocolate have failed [106]. Moreover, serotonin and magnesium are relatively high in chocolate, and these are thought to prevent migraine attacks [106]. Chocolate affects brain oscillations, increasing alpha and beta, and decreasing theta and delta activity [107]. Garlic oil has been shown to reduce the amplitude of KCl triggered spreading depression in rats, and this is possibly due to an effect on astrocytes [108].

There have been questions about the reliability of self-reported triggers—only a few people (3/27) could precipitate a migraine attack from their self-reported triggers [109]. It may be the case that reported triggers are not causal, merely correlated with the migraine attack. Theoretically, it may be more useful to look at triggers that can induce migraine more reliably; however, there are obvious difficulties with this approach. Visual triggers have however been successfully demonstrated to be capable of inducing migraine [110].

### 4.2. Sensory Triggers

Sensory triggers may be more specific to migraine compared to other headache disorders. Stress and lack of sleep were triggers common to both migraine and tension-type headache, whereas sensory factors such as weather, smell, smoke, and light differentiated between migraine and tension-type headache [102]. Similarly, both control and migraine groups commonly reported stress and tiredness as headache triggers; however, 45% of those with migraine, and only 6% of controls reported visual triggers [103]. Visual stimuli included flickering lights, striped patterns, and also computer screen use, reading, bright colours, and optic flow stimuli. Interestingly, light was reported as a trigger in a relatively high percentage of the younger individuals with migraine (10–19 years) [111].

Flicker has been shown to elicit headache in both migraine and control groups, although those with migraine reported experiencing more severe symptoms. This study used 5 Hz stimulation for 5, 10, 15, 25, and 35 min, with 15 and 25 min of stimulation leading to the most intense headaches. As the longest period of stimulation did not lead to the most intense headaches, the author suggests this may indicate habituation to the stimuli [112].

### 4.3. Modelling Triggers

Migraine triggers vary widely, seem to be idiosyncratic, and are unreliable, making it difficult to link triggers to models directly. However, there are some more common triggers, such as stress and sleep deprivation, which may give insights into the state of the brain, e.g., in terms of K+ levels. It seems that, in general, sensory stimulation is more specific to those with migraine compared to other types of headache, although this is not a rule. Repetitive light stimulation has been shown to have an effect on glutamine levels in those with migraine—baseline levels were elevated in those with migraine compared to controls and reduced with repetitive photic stimulation in those with migraine only [95]. Lowered glutamine levels might be expected to *decrease* excitability, as they are the precursor to glutamate, so it seems counter-intuitive that there would be a reduction in excitability to precipitate an attack. However, as previously mentioned, glutamine can also be the precursor for GABA [96], so the effects of photic stimulation may not only affect excitation, but also inhibition levels.

In addition to changes in neurotransmitter levels, oscillatory neural responses are affected by repetitive visual stimulation [113]. This can be a link to both the models, which have been used to represent oscillatory brain activity and to brain activity during migraine aura. Due to the spontaneous and fleeting nature of the attack, recordings during the aura phase are relatively rare; however, some have been achieved, and the evidence will be discussed in the next section.

## 5. Linking Models to Neural Activity during Aura (and Headache)

In order to link theoretical models to migraine in human observers, it would be useful to use recordings of brain activity, such as electroencephalolgy (EEG) and fMRI. There are reports of EEG differences in migraine between attacks (interictally), although this is not sufficient for use as a diagnostic tool [14]. Abnormal interictal EEG is more likely in those with aura compared to those without, and the most common abnormalities were slow waves and spike activity [114]. A review of the interictal literature in EEG in migraine reported that the most commonly reported findings include increased slow wave, theta, and delta power, although the literature is rather mixed [115]. Some authors found reduced alpha frequency between migraine and control participants [116,117]. Differences in alpha power have been reported to be related to migraine history (those with the longest history of migraine, approximately 5 years in a paediatric sample) [118], and also to fluctuate with proximity to the attack [119]. There have in addition been reports of asymmetry of alpha power between the hemispheres [120,121]. There are also reports of interictal increases in power in beta band (12–20 Hz) oscillations [117]. Interictal EEG shows increased theta band power compared to controls in a group including both MA and MO participants [122].

Repeated testing at several points during the migraine cycle has also been conducted using electrophysiological methods. Sand et al. [123] reported increased P1N2 amplitude before attack in a mixed migraine group, and Sand et al. [124] reported increased N1P1 and P1N2 responses specifically in those with migraine aura, which increased before the attack compared to interictally. Shibata et al. [125] reported increased N1P1 amplitude in MA shortly after the attack. Studies investigating habituation of VEP responses found reduced habituation in migraine interictally, but this seems to normalise by increasing to more control-like levels immediately before the attack [126,127]. Evidence has shown that a measure called the “Brain Engagement Index” correlates with the proximity to the migraine attack, peaking before the attack (preictal stage) and reducing afterwards. The Brain Engagement Index was identified as the frequency of occurrence of an individual-specific template of ERP activity in the delta band (1–4 Hz) [128]. This is important as delta band suppression is the EEG correlate of depolarisations measured using intercranial recordings in those with traumatic brain injury [35].

In order to understand the aura itself, and to link to models of spreading depolarisation and depression, recordings are needed during the attack phase (ictal recordings). There are difficulties in recording brain activity during migraine attacks, due to their unpredictable and short-lived nature. However, some recordings have been made, generally either recording those with very frequent or chronic attacks or by inducing an attack.

### 5.1. Slow Waves

One of the earliest studies into EEG in those with migraine studied 51 participants in varying phases of the attack—interictal (between attacks) in some, but also some recordings were made during the aura phase. One such study recorded individuals with basilar-type migraine (BAM), a less common variant of migraine with aura [129]. BAM originates in the brainstem of both occipital lobes and is commonly accompanied with vertigo and lack of co-ordination [130]. Thirty of the 51 were shown to have “abnormal EEG”, although the record lacks details on the abnormalities. Slow-wave (5–8 Hz) abnormalities were reported, and these were exaggerated during the aura in some individuals. However, in other individuals, there were normal resting states and no change in EEG activity reported during the migraine attack phases. Activity seems to be lowered during the main headache stage [129].

Other researchers have also reported pronounced slow-wave activity during an attack in the posterior areas of the brain in those with BAM [131], specifically in the theta band [132]. Soriani et al. [133] reported “diffuse and continuous” reduced alpha, but increased beta band activity, as well as posterior slow waves. After the onset of the headache, there increased activity has been shown in the delta/theta bands in children with migraine, and no abnormalities were found interictally [134]. Other researchers have reported the band to be lower still, with diffuse slow activity in the delta-subdelta range (0.5–2 Hz) in children 4 h after the onset of the attack [135]. All of the participants had normal or improved EEG 4 days after the attack, again suggesting the EEG normalises interictally. In the case of one paediatric participant who experienced migraine aura without headache, EEG recording made the day after the attack showed there to be left occipital slow waves. Additionally, magnetic resonance (MR) perfusion showed hyperperfusion (increased blood flow) over parietal-occipital areas. These changes returned to normal 3 days after the attack [136].

Hyperventilation has been used to elicit migraine attacks, and has resulted in changes to theta and delta band activity; specifically, the delta band changes were bisynchronous slow waves (2–3 Hz) recorded over frontal electrodes [137]. The authors did not include details of the photic stimulation used, so it is unclear why this would not have affected the EEG recording [137]. A PET scan of a spontaneous migraine aura showed there to be spreading hypoperfusion (reduction in blood flow) with time [138]. Migraine aura has also been induced by injection of Xenon (Xe) in the carotid artery. This was successful in 9 out of 13 participants in a study by Lauritzen et al. [139]. Low blood flow was demonstrated on the same side as the injection, starting in posterior areas and spreading through the cortex. However, this low blood flow did not cross the sulcus [139], an attribute of migraine aura that has been modelled successfully as being due to the gyrification of the cortex [64]. Participants who experienced an attack after the Xe injection showed hypoperfusion in posterior areas of the brain, including the occipital, posterior parietal, and posterior temporal areas. Interestingly, rCBF remained unchanged in those who did not experience an attack after the injection [140]. Hypoxia has been used to trigger migraine attacks [141], and hypoxia results in K+ and is related to the spreading depression in animal models [142]. This indirectly links excitability, spreading depolarisation, and depression, as well as electrophysiological activity.

Lee et al. [143] reported several EEG recordings in a case study of hemiplegic migraine (which included confusion and motor aphasia). Hyperventilation and photic stimulation did not affect EEG in this patient, and unusual EEG activity “diffuse slowing” was seen after sleep deprivation. During the recording of sleep activity, POSTs (positive occipital sharp transients) were seen. A large review of EEG studies reporting POSTs found these to be more common in younger individuals and more likely to be accompanied by EEG abnormalities (including slowing and epileptiform activity) compared to controls [144]. Future work investigating POSTs and activity after sleep deprivation may be particularly useful in understanding migraine; however, there are too few controlled sleep trials involving those with migraine to form any conclusions on this at present.

Other authors have reported changes to electrophysiological activity during recordings of visually-induced migraine aura. Bowyer et al. [110] recorded MEG during the migraine aura either from spontaneous (four individuals) or induced (eight individuals) attacks. The attack was induced using black and white chequers reversing at 8 Hz. Those with spontaneous aura showed activation in the right occipital-temporal/parietal region. Those with induced aura showed activation in the primary visual cortex, left occipital and right temporal areas. Direct current (DC) shifts were taken as a measure of activation, and DC shifts were only seen in those with migraine, not in the control group. DC shifts are an overall increase in the amplitude of the measured response and can be indicative of 0.1 to 0.2 Hz slow potentials [145]. These DC shifts were suggested to be indicative of extracellular potassium accumulation and the accompanying spreading depolarisation and subsequent depression [110]. In the cat, negative DC shift is related to membrane depolarisation, which is linked to pyramidal cell activity [146]. This is important as pyramidal cells have been suggested to be a possible biological implementation of the excitatory component of reaction–diffusion models, e.g., [50].

### 5.2. Beta Band Oscillations

Kanai et al. [97] showed that electrical brain stimulation in the beta range (14–22 Hz) makes it easier to elicit flickering phosphenes. How readily phosphenes are elicited is generally considered a measure of cortical excitability [147,148]. During the interictal period, after 30 s photic stimulation at frequencies of 2, 4, and 6 Hz, the beta band amplitude (recorded over temporal areas from channels T3–T5) of those with migraine was found to be increased compared to controls, whereas in those with epilepsy, the alpha band amplitude increased with flash stimulation. Unlike those with epilepsy, those with migraine showed no differences in the power spectral density (PSD) in the absence of flash stimulation [149], showing that the visual stimulation is needed to see differences in migraine.

### 5.3. Alpha Band Oscillations

There are reports of reduced alpha activity (7–13 Hz) contralateral to the aura [150]. Hall et al. [151] reported an MEG recording during the spontaneous aura phase of one individual. This recording showed alpha band desynchronisation (a reduction in alpha power) in extrastriate and temporal areas during the time when the observer reported seeing scintillations and lasting approximately 5 min. Afterwards, the MEG showed gamma band desynchronisation, for the next 16 min, over the inferior temporal lobe. Investigating the EEG at different stages of the migraine attack, Seri et al. [152] reported decreased occipital alpha power during an attack in a childhood migraine, which was contralateral to the aura hemifield. This was followed by an increase in delta power over bifrontal areas, which spread to the posterior-temporal and occipital areas during the headache. Finally, EEG was normal when recorded interictally. The decrease in alpha contralateral to the hemifield may simply be the reduction in alpha that is seen when an observer views a stimulus—reductions in alpha power have been reported in the case of hallucinations (for a review, see [153]). The normal EEG recorded interictally is typical, and a reason why there is no EEG biomarker for migraine. The increase in delta power seems to be a common theme in EEG recorded during the headache phase of the migraine attack, and this may have significance—in individuals with traumatic brain injury, there has been shown to be an EEG correlate, in the delta band, of intercranially measured spreading depolarisation [35].

### 5.4. Linking Electrophysiology to Reaction–Diffusion Models—Oscillations

It may be possible to link oscillatory activity recorded at the scalp to models of migraine aura, in particular for the alpha band. Whilst this was considered the idling rhythm of the brain for a long time, it is now thought that these oscillations have an important role in the inhibition of incoming responses [154]. An individual’s alpha band oscillations are thought to act as a “window of excitability” [155]. An incoming signal that coincides with the trough of the alpha oscillation is more likely to be perceived compared to one that coincides with the peak [156]. Alpha band oscillations are thought to be generated in the LGN, with the activity of bursting neurons being synchronised at gap junctions. This results in “relay-mode” spiking—one group of neurons spiking at the peak of the oscillation and the other group at the trough of the oscillation [157]. Finally, the interneurons provide a cyclic form of suppression to result in alpha band oscillations, which are transmitted through thalamo-cortical neurons into the later visual areas. It is thought that the interneurons and GABA in particular have a relationship with alpha band oscillations, as a recent study showed a positive relationship between alpha band peak frequency and GABA levels [158].

When flicker is synchronised to an individual’s alpha band oscillations, EEG research has shown that patterns of activity consistent with travelling waves take 2–5 s to emerge, compared to 10–15 s for trials that are not synchronised to the alpha band oscillations [159]. These travelling waves translate into visual hallucinations such as circles, spirals, and grid patterns [159]. There are several reports of visual illusions being elicited by flicker [160]. Illusions are elicited at frequencies specific to the individual, as well as the harmonics of the critical frequency [161]. These hallucinations are also able to be elicited with eyes closed [162], although the strength of the visual stimulus will of course be greatly reduced by the eyelids. It is thought that because the alpha band oscillations are greater with eyes closed, the alpha band may be associated with the hallucinations. However, the flicker rate of the shimmering fortification spectra has been estimated to be around 18 Hz [163], which is the range of beta band oscillations.

In order to measure the effects of light stimulation without a specific pattern, a “Ganzfeld” flicker has been used. This essentially incorporates special “glasses” to obscure the visual scene whilst still allowing light through, similar to putting half a table-tennis ball over each eye. Ganzfeld flicker stimulation in non-clinical populations results in radial and spiral hallucinations at lower (5 Hz) and higher (17 Hz peak) frequencies, respectively [164]. When images of these hallucinations were shown to observers, EEG amplitude increased at 4–6 Hz for the radial and 11–21 Hz for the spiral patterns, linking the hallucination and the frequency of stimulation [164]. Hallucinations induced by flicker in non-clinical populations peaks at 11 Hz, and this has been directly modelled using a reaction–diffusion-based system of inhibitory and excitatory cell banks (based on the work of [60], which were then passed through a banks of Gabor filters (an energy model) to detect motion [165]. These models show the relationship between the stimulation frequency and the resulting hallucination. In the stable resting state, in the absence of stimulation, the model displays oscillations at around 13 Hz [60]. This is determined by the set of parameters chosen, including the spatial spread (diffusion term) of the inhibitor being greater than the activator. Importantly, in this model, there are also time constants for the inhibitor and the activator, which determine the period of the whole system’s oscillations. In this case, the time constant for the inhibitor is two [60] or three times [164] that of the activator. Lower-frequency stimulation (around 10 Hz) results in hexagonal patterns of cortical activation, which would correspond to chequered hallucinations. Higher frequency stimulation (around 15–20 Hz) would result in stripes of cortical activation, which would result in hallucinations such as radial patterns and spirals [60]. These studies include how it is possible to model the hallucinations as a result of the systems ongoing oscillations in combination with a repetitive input.

Repetitive visual stimulation, or photic driving, has been of interest in EEG migraine research for a long time, most commonly as an attempt to provide a diagnostic tool that does not rely on self-report. A review of the literature in the 1990s showed that the analysis of photic driving responses is not sensitive or specific enough to be used as a diagnostic tool in migraine; however, there was evidence of abnormalities in the photic driving responses in those with migraine above 16 Hz [166]. Importantly, the authors noted that at the time this had not been linked to the migraine aura, and more recent work has suggested that there is no link to migraine aura [167]. A more recent study showed that compared to control groups, both MA and MO showed increased photic driving response for a 2cpd pattern flickered at 10 Hz, with MA showing a higher response compared to MO [168]. A 2cpd stimulus flickering at 7.5 Hz elicited a greater response in MA compared to MO [169]. Curiously, those with MA showed a weaker response to photic stimulation compared to MO for frequencies of 5, 8, 15, and 20 Hz [170]. The lack of conclusive findings makes it difficult to draw firm conclusions about the role of photic driving. It is important to note that repetitive visual stimulation can entrain neural responses [171] or at least evoke repetitive responses (steady-state visual evoked potentials) [172], and the amplitude of these may depend on the frequency of the ongoing oscillations. Future work should first measure the ongoing oscillations, as this is likely to impact the amplitude of the response to repetitive visual stimuli. However, the question of how exactly photic stimulation relates to susceptibility to migraine aura is still an open one. Repetitive stimulation can excite the cortex, and entrain oscillations, so these might be good candidates for possible mechanisms.

### 5.5. Linking Electrophysiology to Models—Connectivity

It is especially important to consider the ongoing oscillations as these relate to functional connectivity of the brain. It must be noted that recordings of brain activity (whether using EEG or fMRI) are not generally on the same scale as the models—models work on the level of cells, or groups of cells (e.g., [50,63]), whereas EEG recordings do not have the spatial resolution for this. However, recent work has shown that by using networks of reaction–diffusion equations, the synchronisation properties of epileptic seizures could be modelled [173]. The synchronisation properties were dependent on global aspects of the network, including the strength of the connectivity between regions. This is important as there is already work available on the effective connectivity in the migraine brain, estimated using EEG after photic stimulation.

Estimates of functional connectivity after photic stimulation can discriminate MA and MO. After flash stimulation of 9–27 Hz, the amplitude of the response was higher in MA and MO compared to controls over the occipital and parietal channels [174]. Additionally, functional connectivity (Granger causality) in the beta band differentiated between MA, MO, and controls [174]. Granger causality is estimated by taking the autoregression of a time series (x) to predict future activity from past activity. This is compared to the autocorrelation from a second time series (y). The error in the prediction from the two time series together is compared to the error in the prediction for the time series x alone. Effective connectivity (based on Granger causality) was higher in MA compared to MO in alpha and beta bands after 5 and 10 Hz photic stimulation using 0.5 and 2cpd patterns [175]. Specifically, activity was spread over a larger extent over fronto-central and occipital regions in MA [175], and information was estimated to be flowing posterior to anterior [176]. Granger causality increased with laser stimulation (to cause pain) in those with migraine [177], and Granger causality was increased in migraine in specific areas associated with pain processing [178]. Overall, there is evidence to suggest that information spreads more readily in migraine from posterior to anterior regions, specifically in alpha and beta bands. This has implications for the models; those areas with increased responses to visual and painful stimuli are propagating information to other regions, resulting in increased responses. It may be possible for models to capture this aspect of rate of information propagation interictally in the migraine brain, which may relate to susceptibility to travelling waves, and the susceptibility of the cortex to precipitating factors.

In summary, the migraine aura seems to be characterised by oscillatory differences including pronounced slow-wave activity, reduced alpha power, and increased beta power. Factors thought to elicit the migraine attack including hyperventilation, sleep deprivation, and photic stimulation have been related to oscillatory differences in EEG. In studies with multiple recordings, it appears to be the case that activity normalises after the attack, suggesting that these complex changes are indeed linked to the attack itself. Importantly, recent research has shown fluctuations of oscillatory brain activity (in delta and beta bands) in proximity to migraine attack [179], linking these oscillations to the attack itself more convincingly. Reaction–diffusion models can be used to model the oscillatory behaviour of the system, and in particular, recent work has included a Laplacian function as a diffusion parameter to represent the functional connectivity of the network [61]. Functional connectivity can be estimated from EEG and fMRI recordings, so this will help to link the theoretical models and the oscillatory behaviour shown experimentally.

## 6. Linking Models to Psychophysical Evidence and Signal Detection Models

Behavioural methods can also be used to give indirect estimates of the state of the brain and possible susceptibility to aura. Migraine has been identified as primarily a disorder of sensory processing [28]. As such, some reliable differences in visual perception have been demonstrated in migraine, particularly migraine with aura [180]. These differences show that there are some fundamental differences in the operation of visual processing mechanisms in migraine with aura [180,181,182,183], which may be linked to the susceptibility to cortical spreading depression and the hallucinatory experience of visual aura.

Basic visual functions have been demonstrated to correlate with GABA levels, for example, increased contrast sensitivity with higher GABA levels [184]. Contrast sensitivity is a measure of the overall sensitivity of the visual system to detect visual targets and is generally found to be lower interictally in migraine, despite the hyperexcitability associated with the condition. There is some evidence that sensitivity is at typical or enhanced levels for small, centrally presented stimuli [182,185,186,187] when presented without a noise mask, and the overall reduced sensitivity may be linked to patchy impairments of sensitivity across the visual field [188].

Orientation discrimination performance has also been shown to relate to GABA levels [189], measured using magnetic resonance spectroscopy, as well as gamma band oscillations [190]. Orientation discrimination has been shown to be poorer in migraine compared to controls [191,192]. The findings are mixed, however, as other research has shown no impairment in orientation discrimination [193], although these authors did show some tentative evidence that the number of migraine aura experienced by participants may relate to orientation discrimination impairments.

The relationship between psychophysical tasks and neurotransmitters is not straightforward. For example, surround suppression of motion and binocular rivalry have been shown to relate to GABA levels measured using magnetic resonance spectroscopy [194]. However, contrast surround suppression of a moving stimulus is *increased* in migraine [195]. Yazdani et al. [196] assessed two forms of surround suppression, one motion-related, one contrast-related, and found no correlation. This shows that the two types of surround suppression must have independent mechanisms, and so only one, or neither of them, relate to GABA levels. It is possible that only the motion-based surround suppression reflects levels of GABA. A recent study showed no difference between those with migraine and controls in terms of GABA levels, and no difference in terms of performance on binocular rivalry performance [94]. There was also no relationship between either combined glutamate and glutamine or GABA levels and binocular rivalry performance [94].

Glutamate concentration levels can affect visual task performance. Those with higher levels of Glx show lower motion perception thresholds (more sensitive to motion stimuli) as well as greater fMRI responses [197]. This is again at odds with the migraine literature—those with migraine are thought to have higher levels of glutamate [93]; however, a robust behavioural finding is poorer motion perception performance (see [180] for a review). The type of illusory motion perception after viewing a moving stimulus depends on the duration of the priming stimulus as well as individual differences: it could be in either the same direction (assimilation) or the opposite direction (contrast). Using MR to estimate glutamate and GABA levels, the switch between motion assimilation and motion contrast was found to depend on glutamate concentration in the dorsolateral prefrontal cortex. There was no relationship between type of motion perceived and glutamate or GABA levels in either V1 or area MT [198]. This suggests glutamate is not involved in the perception of motion per se, but instead the switching of the percept in the higher-order areas.

Linking the behavioural data to specific neurotransmitters directly is difficult due to the complexity of the system. However, the behavioural data have been interpreted on a more abstract level using signal detection models. For example, some of the most reliable differences in sensory processing in migraine with aura have been found in visual masking. Studies of global motion and form, in which observers detect a global pattern, distributed across many stimulus elements, in the presence of noise, has generally been found to be poorer in migraine [182,183,199]. Tibber et al. [200] showed that poorer performance in a mixed migraine group points to a deficit in the ability to detect a target signal while excluding a distracting mask, rather than a more general impairment in the encoding of visual stimuli. These results have been interpreted using signal detection models of visual processing, which specify the gain control on the strength of the initial encoding of visual stimuli and the presence of noise [182,183,200]. This noise may be additive (independent of the magnitude of the stimulus response) or multiplicative (increasing with the size of the response to the signal). O’Hare and Hibbard [180] concluded that the best account of the hyperexcitation found in migraine, and the psychophysical differences summarised above, is one in which there is an increase in background noise, combined with an increased gain in visual encoding. Both of these characteristics will predispose reaction–diffusion models to the types of hallucinations found in migraine aura, by increasing the likelihood of a short burst of hyper-excitation that would then trigger the larger wave of depolarisation, and are consistent with the physiological differences that are associated with cortical spreading depression.

### 6.1. Differences in Performance over the Migraine Cycle

In order to understand migraine aura, it is important to expand knowledge into how performance changes over the course of the migraine cycle. Studies have shown correlations with visual performance and self-reported time since the last attack, e.g., [201].

Studies investigating just a few time points have also shown differences. Visual field deficits in those with migraine compared to control groups, although quite consistent across time, exhibit some local deficits that may be more pronounced one day after attack [202,203]. When there was a longer (on average) delay after the attack itself, no differences in visual field measures were found, although there were differences in electrophysiology rather than psychophysical measures; specifically, steady-state visual evoked responses were higher post-attack compared to interictally [204].

Behavioural studies involving multiple testing sessions have also been conducted. McKendrick et al. [205] showed that ictal centre-surround suppression (thought to be indicative of cortical inhibition) is stronger in migraine compared to control groups, but this decreases in the days surrounding the attack. Cycle effects have been reported using after-images—after-images are shorter in those with migraine compared to controls, but the duration increases through the migraine cycle, peaking on the day of the headache itself [206]. Subjective unpleasantness ratings for visual and odour stimuli were greater in the headache days compared to interictally, and this was related to increased connectivity in the hypothalamus and brainstem [207].

The gold standard for measuring cycle effects is repeat testing throughout *several* migraine cycles, which involves an intensive testing schedule for a long period of time. Shepherd [208] showed interictal global motion performance was poorer in migraine compared to control groups and that performance improved in the 2 days before and the 2 days after the attack itself. There were no differences in orientation discrimination between groups, and no reliable cycle effects in orientation discrimination performance.

Neither signal detection models nor reaction–diffusion models currently consider the differences in the migraine cycle. This is an important step for future research in order to understand the disorder more fully and be able to devise therapy.

### 6.2. Linking Back to the Models

In order to make links between models of migraine aura and psychophysical behaviour and signal detection models, it might seem sensible to start with the parameters that make the network susceptible to spreading depolarisation and subsequent depression. There are several parameters that affect the susceptibility of reaction–diffusion models to travelling waves. One of the main ones is the relative diffusion rates for the activator and the inhibitor. In some of the more complex models, there are non-linear equations for the reaction component, which can also effect the susceptibility to travelling waves, e.g., [41]. Given the number of possible neurochemical mechanisms that could possibly be represented by the different parameters, identifying the correct ones in those with migraine is not trivial. This might leave the reader wondering if there is any point to all these models at all. The issue here may be the level of abstraction: instead of thinking at the level of neurotransmitters, it may be more worthwhile to look at the level of measurable behaviour to link this to the models. There are possibilities for doing this in terms of EEG estimates of functional connectivity, for example. This has been started in recent work in epilepsy and may be a fruitful area when considering migraine [149].

It may also be possible to link the models of aura to visual performance from these individuals, throughout the migraine cycle if possible. For example, signal detection theory has been used to account for behavioural data with some success in migraine, e.g., [182,183]. It has been suggested in the case of those with migraine aura that there will be a particular susceptibility to external stimuli due to increased internal noise levels, thought to be due to increased spontaneous neural firing rates [180]. One outstanding question in migraine aura is what initiates the migraine aura in the first place. One possibility is input to the system, in the form of triggers; however, triggers are idiosyncratic and have been demonstrated to be unreliable [109]. In the original reaction–diffusion model, the initiation of the pattern formation was due to spontaneous fluctuations of activity [46]. This could provide an important bridge between reaction–diffusion systems modelling aura and signal detection theory modelling behaviour—both would predict increased levels of spontaneous firing, which would make the initiation of an attack (all other things being equal) more likely.

In order to link models to perceptual performance, it is important to understand which elements of the models relate to perception. It has been suggested that the output of the pyramidal cells relates to our perceptual experience [50]. The idea of where perception happens is beyond the scope of this review, but it is important to note that the model outputs generally plotted are the excitatory system at that particular time point (e.g., [54]), which could be the output of (groups of) pyramidal cells. Neural oscillations, specifically alpha band oscillations, have been directly linked to perceptual performance. For example, observers are more likely to detect a stimulus if its arrival coincides with the trough of the alpha band oscillation (low alpha power); however, the arrival of the incoming stimulus has the effect of increasing phase locking (synchronisation) of the alpha band oscillations and therefore increasing the resulting ERP component [209,210,211]. Those with migraine aura also show an increase in ERP response amplitude to incoming stimuli compared to controls [125,212,213], which would be consistent with this idea; however, they show *increased* lower alpha band (8–10 Hz) power in the resting state compared to controls [214]. These recordings were all taken interictally, and so given that the alpha band reduces during the attack [151,152], it is essential to look at cycle effects in the EEG before drawing firm conclusions.

This is a relatively unexplored area, but it might help to use these models of the migraine aura in combination with experimental results on EEG behaviour during an attack and behavioural performance during the migraine cycle to help understand how attacks are initiated, which may help with preventing them.

## 7. Discussion and Unresolved Questions

This review outlines reaction–diffusion models of the classic features of migraine aura, the zig-zag fortifications and the scotoma. These reaction–diffusion models of inhibitory and excitatory interactions between networks of neurons are flexible abstractions that help understand the dynamics of the cortical spreading depolarisation and depression, respectively. Factors predisposing models to cortical spreading depolarisation and depression are (1) the balance between strength and spatial range of excitatory and inhibitory interactions and (2) the likelihood of occurrence of spontaneous hyperactivity, which could trigger a wave of depolarisation. Direct links to electrophysiology are difficult due to the lack of experimental data during the attack; however, neural oscillations may be a fruitful area of investigation as these relate to functional connectivity of the network and the excitability of the brain, which may relate to the terms of the models. Additionally, neural oscillations have been shown to fluctuate over the migraine cycle, linking them to the disorder. Recent advances in wearable technology may open possibilities for investigating oscillatory behaviour and functional connectivity estimates throughout the migraine cycle, which would be able to directly link to the models. For example, wearable technology has been used to estimate electrophysiological activity at different stages of the migraine cycle [179,215].

Psychophysical methods can also be used to determine the relevant parameters for the models, as these provide indirect estimates of cortical excitability. For example, signal detection models accounting for perceptual performance predict increased noise and gain in the migraine brain. These may relate to reaction–diffusion model parameters, e.g., internal noise levels may relate to the initial random fluctuation in activity that initiates the travelling waves in the models. Importantly, perceptual performance varies over the migraine cycle, which would be expected if the underlying processes relate to the disorder itself.

### 7.1. Arguments against Cortical Spreading Depression as the Mechanism for Migraine

Importantly, there are several arguments against describing migraine aura through cortical spreading depression and models thereof (see [216,217] for detailed discussion). Firstly, cortical spreading depression has been robustly recorded for patients who have experienced a stroke or head trauma [35,218] where it is possible to obtain continuous electrocortical recording via cortical electrodes or subcortical electrocorticograms. In contrast, to date, there is no electrophysiological evidence of spreading depression during a human migraine attack.

There are also questions about how cortical spreading depression accounts for more complex aura and hallucinations. For example, bilateral visual aura, which are experienced across both hemispheres, are not explained by cortical spreading depression, since propagation requires contiguous grey matter and does not cross the corpus callosum [217]. Since spreading depression only propagates in the hemisphere of origin, it is also argued [216] to insufficiently account for the bilateral pain experienced by 40% of people with migraine.

Thus, it is important to note that the models are an oversimplification of the diversity of migraine aura, and at present, the models focus on the typical, simple geometric hallucinations. There are more complex hallucinations such as hallucinations of people and objects, e.g., [9], which tend to be idiosyncratic and less common than the typical zigzag lines, flashing lights, and scintillating scotoma [8,9]. These more advanced illusions are not included in the current models and complex patterns may be a result of a secondary involvement of higher visual areas of the brain, such as those areas specialised for face and object recognition.

Migraine is a multi-faceted disorder, and there is also variation in each individual’s attacks. The models present a simplification of one possible set of events, and do not take this variability into account. Models currently limit themselves on the propagation of the travelling wave, rather than inferences about the possible hallucinations if it were to spread to higher-order visual areas. However, recent work by Kroos et al. [219] has generated individual-specific reaction–diffusion models using the individual’s MRI data. This model successfully recreates the diffusion of water as an approximation of electrical conductivity, and importantly, the shape of the individual’s cortex is incorporated in modelling the spreading depression for that individual. This is important as it is the first step to modelling the individual-specific variation in aura.

### 7.2. Can We Infer Clues to Aura Susceptibility from the Models?

One outstanding question is whether there are behavioural and electrophysiological clues to aura susceptibility, and this might be able to be predicted from the models. For example, alpha band oscillations relate to the inhibition of responses, which may facilitate the spread of the migraine aura. Increased internal noise may also relate to the susceptibility to migraine aura, and this could be measured using behavioural techniques. Predictions about internal noise levels could be made based on varying model parameters, generating clear, testable hypotheses. Given the difficulty of direct physiological measures during the migraine aura, it may be possible to use psychophysical measures with well-established hypotheses to provide a better route to understanding altered sensory processing.

### 7.3. How the Models Account for Pain

Another unresolved element is the link to the headache and pain of migraine. It is common to experience migraine headache without aura, and it is also possible to experience aura without pain. Some approaches consider migraine without aura as intermediate, between attacks with aura and no attack. The reaction–diffusion models are limited to the aura and do not explicitly account for pain. However, Kroos et al. [220] recently applied a reaction–diffusion equation to model spreading depression for five single case studies of patients with migraine. Simulated spreading depression was personalised for each patient, matching their symptoms during a migraine attack to the wavefront propagation based on acquired MRI data. In the simulation, the spreading depression reached the primary and secondary somatosensory cortex, specifically the topographical area related to the trigeminal nerve, and pain processing prefrontal areas. The authors suggest that the propagation of extracellular K+ may elicit the headache by activating afferent pain receptors [220]. This is important as the spreading depression is linked to the activation of the trigeminal neurons that are involved in mediating lateralised head pain [217]. Future work to link the spreading depression and the head pain could use reaction–diffusion models as a basis.

### 7.4. Long-Term Effects and Stroke Risk

There is also the possibility of long-term effects of migraine. It has been suggested that repeated migraine attacks increase the likelihood of subsequent migraine attacks (for a review, see [221]). Frequency of attacks can increase in some individuals, eventually even progressing to chronic migraine, and this may be due to sensitisation of the system from repeated spreading depolarisation and depression mechanism [222]. Although migraine is thought to be benign [223] and the attacks fully reversible [224], there is some evidence of possible long-term damage from regular attacks [2,225,226,227]. There is also an increased risk of stroke in those with migraine aura [228]. Stroke is also thought to be a disorder as a result of spreading depolarisation [218,229], so this is an important avenue to be explored in terms of models of spreading depolarisation and depression.

### 7.5. Age and Sex Differences

The majority of the literature in this review is focused on adults with migraine aura, and therefore, the conclusions must be restricted to this population. However, it is important to consider those with paediatric migraine as well. The prevalence of paediatric migraine overall is around 8%, and around half continue to experience migraine into adulthood [230]. However, the prevalence of paediatric migraine aura is much lower, estimated to be around 1.6% [231]. The clinical characteristics are similar to those of adults, with the majority experiencing visual aura, and this is stable across the age bands investigated (under 6, 7–10, 11–14, and over 15 years) [232].

There are also sex differences in migraine, with migraine being more prevalent in females than males, and this interacts with age. Across the age range of 3 to 85+ years, migraine prevalence is estimated to be 17% in females compared to 8% in males, with the ratio females to males being the greatest between the ages of 20–40 [233]. However, under the age of 10, female and male prevalence was found to be similar [233]. Research has shown the median age of onset is estimated to be mid-twenties for both men and women, suggesting that puberty is not a factor in determining migraine [234].

Hormonal changes have been thought to be the case of the increased prevalence in migraine in females compared to males. However, there are differences between migraine with and without aura—attacks without aura are more likely with the withdrawal of oestrogen, whereas migraine with aura attacks are more likely when there are high levels of oestrogen, for example pregnancy [235]. Diary studies have shown that migraine occurs around the onset of menstruation in those with migraine without aura, but not migraine with aura [236], and higher peaks in oestrogen levels across the menstrual cycle have been found in migrane with aura [235]. Additionally, systematic reviews of the literature have found that migraine without aura seems to be more affected by the transition of menopause compared to migraine with aura, which seems to be more stable [237,238]. Research has shown that migraine aura is not related to menopause [239].

The current models do not consider any age-related or sex-related differences at present, but this could potentially be incorporated in future. For example, it has been proposed that visual arousal might be increased around the peak in oestrogen levels prior to ovulation [240]. The higher peaks in oestrogen level in migraine with aura might thus be associated with increased excitability in the visual cortex. While some studies have suggested an increase in visual sensitivity around ovulation [241,242,243,244,245,246,247], others have shown no effects on contrast sensitivity [248]. It would, however, be informative to study this directly in cases of migraine with aura.

Interestingly, it has been shown that spreading depolarisation cannot be elicited using K+ in the neonatal rat before the age of 12 days [249]. Understanding the factors that protect the juvenile brain from spreading depolarisation is of interest for the progression of migraine and worth exploring in future modelling work.

## 8. Conclusions

This review has outlined reaction–diffusion models for a general readership, with the aim of improving accessibility to the diverse disciplines involved in understanding migraine. Direct experimental literature supporting these models is sparse, since measurements during the attack are logistically difficult. Ideally, intercranial recordings during the aura under different conditions would provide measures of the cortical dynamics of cortical spreading depolarisation and depression; however, such measures are not possible. In order to treat migraine, we need to know the mechanisms of the aura and, perhaps more importantly, the factors determining susceptibility to attacks. This is where reaction–diffusion models could be useful to bridge the gap between proposed mechanisms and testable hypotheses, since they are able to illuminate the “black box” of what is happening during the aura itself, and develop hypotheses that can be tested using electrophysiological and psychophysical techniques. It would be especially useful to track the changes in model parameters and compare to experimental data across the migraine cycle. With longitudinal behavioural evidence becoming easier to obtain through advances in wearable technology, the theoretical predictions of these models will be testable in the near future.

## Figures and Tables

**Figure 3:**
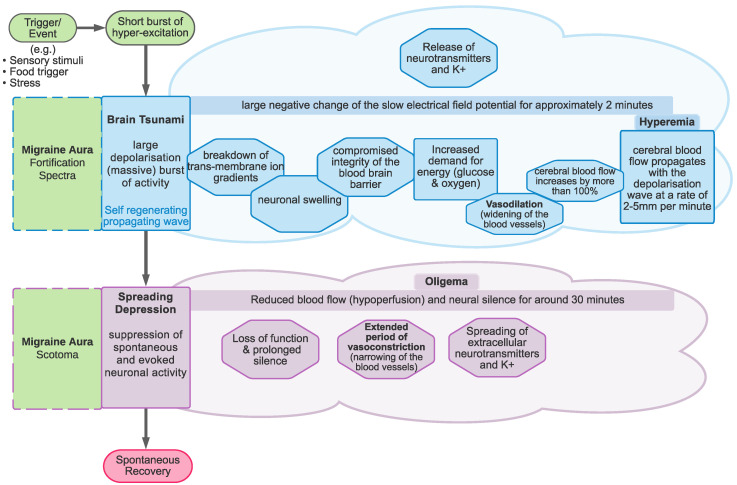
Cortical spreading depolarisation and depression in migraine. Spreading depression and the migraine scotoma start simultaneously with the onset of the negative potential shift of the deplorisation (brain tsunami).

## Abbreviations

The following abbreviations are used in this manuscript:

AC	Alternating current
BAM	Basilar-type-migraine
DC	Direct current
GABA	Gamma-aminobutyric acid
Glx	Combined glutamate and glutamine complex
K+	Potassium ion
MA	Migraine with aura
MEG	Magnetoencephalogram
MO	Migraine without aura
MR	Magnetic resonance
MT	Middle-temporal
NDMA	Nitrosodimethylamine
PSD	Power spectral density
rCBF	Cerebral blood flow
tACS	Transcranial alternating current stimulation
tDCS	Transcranial direct current stimulation
TMS	Transcranial magnetic stimulation
Xe	Xenon
